# The Medical Mission Field

**Published:** 1924-06

**Authors:** 


### THE MEDICAL MISSION FIELD.

"In some rural districts of India," said a speaker at the annual meeting of the Medical Mission Department of the Society for the Propagation of the Gospel, "there is one public health office to 45,000 people." And India does not stand alone in the need of medical men and women. China, South and West Africa, Borneo, Mauritius—these are only some of the countries for which the Society is needing workers and needing them most urgently.

### A Close Alliance.

Sir Humphrey Rolleston, K.C.B., President of the Royal College of Physicians, who took the chair, pointed out the close alliance which existed between the care of the soul and the care of physical ills. He could not sufficiently express his admiration of those noble members of his profession who devoted themselves soul and body to medical missions. They followed the example of Luke, the beloved physician, and scorning ambition and selfish aims gave themselves in a manner to make some of us feel ashamed. The male sex was unhappily far behind in the mission field, and though there was much for women to do there were some places where it was more convenient and perhaps only possible for men to go.

### The Needs of India.

Miss Dora Tickell, one of the Society's Delegation just returned from India, spoke of the great advance in medical work there in the extension of Infant Welfare Centres and other health activities. Doctors were badly required in the towns, but infinitely more so in the country. The people simply could not face a jaunt by bullock carts of some fifteen miles to a station, followed by a railway journey of forty miles or so before they could reach a hospital where they would be unknown and afraid. They needed hospitals close at hand. The best way of meeting the difficulty would be for the Society to establish small dispensaries staffed by Indian medical men and women, linked up with one big central hospital in the charge of a first-rate doctor.

### A Doctor for China.

Miss Grace Crosbie, lately of the American Church Mission in Central China, gave a vivid account of life in a Chinese hospital, where the lady doctor's work began at 6 a.m., often not ending till midnight, and her sole assistant was a young Chinese intern, who, though utterly devoted, was necessarily inexperienced. It was, she said, poor economy to overstrain workers, so that after learning the language and understanding the conditions they were often compelled to return home utterly worn out and unfit for further mission work. But it was not the fault of S.P.G. Dr. Weir, the Medical Secretary to the Society, said that in eleven years only six new men had offered themselves. They hoped that when the effects of the war were no longer felt more candidates would be forthcoming. During 1923 the Society spent upon the missionaries and hospitals the sum of £22,144, and its income amounted to £21,396. It is to be hoped that work of the greatest value and importance will not be allowed to suffer for want of volunteers.

